# The genotoxicity and systemic toxicity of a pharmaceutical effluent in Wistar rats may involve oxidative stress induction

**DOI:** 10.1016/j.toxrep.2015.09.004

**Published:** 2015-09-24

**Authors:** Grace O. Adeoye, Chibuisi G. Alimba, Olanrewaju B. Oyeleke

**Affiliations:** a Parasitology Unit, Department of Zoology, University of Lagos, Nigeria; b Cell Biology and Genetics Unit, Department of Zoology, University of Ibadan, Nigeria; c Ecotoxicology Unit, Department of Zoology, University of Lagos, Nigeria

**Keywords:** Biochemistry, Hematology, Histopathology, Micronucleus test, Pharmaceutical effluents, Rats

## Abstract

There is scarcity of information on the possible mechanisms of pharmaceutical effluent induced genotoxicity and systemic toxicity. This study investigated the genotoxicity and systemic toxicity of a pharmaceutical effluent in Wistar rats. Rats were orally treated with 5–50% concentrations of the effluent for 28 days. At post-exposure, blood, liver, kidney and bone marrow cells were examined for alterations in serum biochemical parameters and hematological indices, histopathological lesions and micronucleated polychromatic erythrocytes formation (MNPCE). The effluent caused concentration independent significant (*p *< 0.05) alterations in aspartate (AST) and alanine (ALT) aminotransferases, superoxide dismutase (SOD), catalase (CAT), malondialdehyde (MDA), total and direct bilirubin and creatinine. There was reduction in red blood count (RBC), hemoglobin concentration (HGB), platelets, percentage hematocrit (HCT), white blood count (WBC) and mean corpuscle hemoglobin (MCH) except mean corpuscle hemoglobin concentration (MCHC), which increased in the treated rats. Histopathological lesions observed in the liver and kidney of the effluent treated rats were thinning of the hepatic cord, kuffer cell hyperplasia, vacuolation of the hepatocytes and renal cells, multifocal inflammatory changes, necrosis and congestion of the renal blood vessels and central vein. MNPCE significantly increase in the bone marrow of the treated rats compared to the negative control. The concentration of some toxic metals and anions in the effluent were above standard permissible limits. These findings showed that the pharmaceutical effluent caused somatic DNA damage and systemic toxicity in rats may involve induction of oxidative stress, suggesting environmental contamination and health risks in wildlife and humans.

## 1 Introduction

There is unprecedented increase in the pollution status of most aquatic and terrestrial environment worldwide, due mainly to wastewater and solid waste discharge from anthropogenic activities. Increase in the production and utilization of pharmaceuticals across the world have caused the pharmaceutical industry to be one of the major source of solid wastes and effluent discharge into the environment [1]. Pharmaceuticals are chemicals used for diagnosis, treatment (cure/mitigation), alteration or prevention of diseases, health condition or structure and function of the body [2]. Solid wastes and effluents generated during pharmaceutical activities are enormous and diverse due to the use of different chemicals during production processes of various drugs. Also, animal production in many countries is increasing in recent time so as to provide adequate food required by the increasing human population. Achievements in animal farming involve the use of drugs like antibiotics, food additives, hormones and parasiticides to boost production. Many of these drugs are excreted as active metabolites or unmetabolised, while others may escape being degraded in the waste treatment plants. These activities along with improper disposal of expired drugs by burning and burying in the soil release hosts of xenobiotics into the environment [3]. While in the environment majority of the pharmaceutical products accumulate in water bodies, aquatic sediments, soil, and biological systems, reaching a biologically active concentration with time [4], [5].

The volume of water used during various drug operational stages contain numerous chemicals and microorganisms which are discharged as pharmaceutical effluent into the environment, and this has been reported to be highly toxic to living organisms mostly due to the presence of salts, surfactants (such as detergents, emulsifiers and dispersants), ionic metals and their metal complexes, toxic organic chemicals, biocides, unmetabolized drugs, toxic anions and microorganisms [4], [6], [7], [8]. In Nigeria, most pharmaceutical industries located in the cities are known to discharge huge volumes of untreated effluent directly into the environment [9], [10]. This is enhanced by ignorance on the part of many Nigerians on the deleterious effects of drugs and many other xenobiotics including microbes present in the effluents on environmental degradation and public health [6]. Moreover, poor enforcement of stringent regulations prohibiting illegal discharge of effluents is not favoring environmental conservation. There are increasing concerns about the adverse effects of these effluents on the ecosystem, wildlife diversity and human health. With respect to this, studies have identified specific chemical components in pharmaceutical effluents to show the deleterious effects on living organisms [3], [10], [11], [12]. This approach has a limitation of providing information on the toxic effects of all the individual components present as mixture of xenobiotics in the effluent. Also the potential synergistic and antagonistic interactions of these xenobiotics in the living organisms were not considered. Therefore, experimental toxicity studies were conducted both in the laboratory and on the field to determine the toxic and genotoxic effects of pharmaceutical effluents as a complex mixture of xenobiotics [4], [7], [8], but most of these studies are limited by their inability to provide possible mechanisms of pharmaceutical effluent induced mutagenicity, genotoxicity and systemic toxicity.

Due to the specific mode of action and the fact that drugs and pharmaceutical compounds are deliberately designed to exert their effects on humans, mammals and other vertebrates, the systemic toxicity and genotoxicity assessment of pharmaceutical effluent using mammalian test model will be more relevant in relating findings to human health. Biomarkers of systemic toxicity (biochemical, histopathological and hematological analyses) in mammals offer better understanding of the possible mechanisms of genotoxicity and tissue damage in the case of exposure to mixture of xenobiotics [13], [14], [15], [16]. However, until now the mode of action of pharmaceutical effluents induced genotoxicity in mammalian system is not well understood. Many of the components (most importantly toxic metals) are known to induce oxidative stress as a major mechanism of chemical induced toxicity [17]. Furthermore, the liver and kidney are sensitive predictor of chemical induced toxicity, due to their ability to metabolize, detoxify, store and eliminate xenobiotics and there metabolites. Hence, they are target organs for xenobiotic induced toxicity [18], [19]. Hence, the need to understand the possible mechanisms by which pharmaceutical effluent may induce liver and kidney dysfunction, and genotoxicity in mammalian system. This study investigated the frequency of micronucleated polychromatic erythrocytes (MNPCE) formation, histopathological lesions in the liver and kidney, changes in hematological indices, and alterations in serum antioxidant enzyme activities, lipid peroxidation and liver and kidney biochemical function parameters in pharmaceutical effluent treated Wistar rats. Some physico-chemical parameters and heavy metal constituents of the effluent were also analyzed.

## 2 Materials and methods

### 2.1 Effluent collection and processing, physico-chemical and heavy metal analyses

Pharmaceutical effluents were collected from a pharmaceutical industry with branches located in Ilupeju, Lagos state and Sango Ota, Ogun state both in Nigeria, into a 10 L transparent plastic container. The pH of the effluent was measured before transported to the laboratory for further processing. An aliquot was analyzed for a number of standard physical and chemical parameters including chemical oxygen demand (COD), total dissolved solids (TDS), alkalinity, hardness, biochemical oxygen demand (BOD), chlorides, sulphates, nitrates, ammonia and phosphates in accordance with the APHA [20]. Seven heavy metals; cadmium (Cd), arsenate (As), copper (Cu), chromium (Cr), iron (Fe), zinc (Zn), nickel (Ni) and manganese (Mn) were also analyzed in the effluent in accordance with standard method [20], [21]. 100 ml of the effluent was digested by heating with concentrated nitric acid (HNO_3_). The resulting mixture was made up to 10 ml using 0.1 N HNO_3_. The concentration of the metals was estimated using PerkinElmer^®^ A3100 atomic absorption spectrophotometer. Another aliquot was filtered with glass wool and Whatmann^®^ No. 42 filter paper to remove suspended particles; centrifuged at 3000 g for 15 min and stored at 4 °C until use for the animal exposure. This was considered as the stock sample (100%).

### 2.2 Animals and experimental design

Male Wistar rats (between 8 and 9 weeks old) obtained from the animal unit, College of Medicine, University of Ibadan, Nigeria, were acclimatized for 2 weeks in the Department of Zoology, University of Lagos animal facility until they attained a body weight range of 126.4–161.8 g. They were maintained in laboratory conditions of 12 h dark and light cycle, temperature of 26.9 ± 6 °C and had access to clean drinking water and standard rodent chow (Ladokun feed Nigeria^®^) *ad libitum*. Guide for care and use of laboratory animals published by the US National Institutes of Health (NIH Publication No. 85–23, revised in 1996), and approved by the ethical committee, University of Lagos for the use of animals in experimental studies was carefully adhered during the study. 5, 10, 20, 30 and 50% concentrations (v/v, effluent/distilled water) of the effluent were selected according to previous study [22], and 0.5 ml was orally administered daily to each of the five rats per exposure group for 28 days [15]. Similar treatment was concurrently given to the negative (distilled water) and positive (cyclophosphamide 40 mg/kg body weight) control groups.

### 2.3 Serum biochemical analysis

At post-exposure, rats were fasted overnight and blood was collected from the orbital plexus using heparinized 70 ml micro-hematocrit capillary tubes into lithium coated serum separator tubes and EDTA tubes. The clotted blood in the lithium tubes was centrifuged at 3000 g for 10 min to separate the serum (supernatant) and stored at −70 °C prior to biochemical analysis. Serum biochemical markers of oxidative stress were measured according to standard protocols: Catalase (CAT; EC 1.11.1.6) activity was measured according to Aebi [23], superoxide dismutase (SOD; EC 1.15.1.1) activity was measured based on Magwere et al. [24] method, while lipid peroxidation was measured as malondialdehyde (MDA) concentrations in accordance with Nichaus and Samuelson [25]. Protein concentration was measured according to Lowry et al. [26]. Serum liver and kidney functional test markers; transaminases were measured according to Reitman and Frankel [27], total and direct bilirubin according to Treitz [28] and creatinine according to Henry et al. [29] using Randox Laboratory (UK) diagnostic kits. The absorbance for all the reactions were measured spectrophotometrically using HAICE^®^, DR 3000 (Germany).

### 2.4 Haematological analysis

Blood collected into the EDTA bottles was analyzed for the recommended hemogram: total erythrocyte count (RBC), hemoglobin content (HGB), percentage hematocrit (HCT), mean corpuscle hemoglobin concentration (MCHC), mean corpuscle hemoglobin (MCH), platelets (PLT) and white blood cell count (WBC) [30] using automated analyzer, (Abbott Hematology Analyzer Cell-Dyn 1700, Abbott Laboratories, Abbott Park, Illinois, USA).

### 2.5 Mammalian bone marrow micronucleus assay

Animals were sacrificed by cervical dislocation and the femoral bones surgically removed for bone marrow micronucleus test [8], [31]. The bone marrow was flushed into Eppendorf tubes using 0.5 ml of Fetal Bovine Serum (FBS). The cells were centrifuged at 2000 g for 5 min and smear made on pre-cleaned grease free slides. Slides were air dried and stained with May–Grunwald and Giemsa stains. They were coded and examined under an Olympus light microscope at 1000× magnification. 2000 cells per rat were scored for micronucleated polychromatic erythrocyte (MNPCE).

### 2.6 Histopathological analysis

Slices of the right lobe of the liver and right kidney from effluent treated and control rats were fixed in 10% neutral buffered formalin. The tissues were dehydrated in ascending order of ethyl alcohol–water concentrations, cleared in xylene and sequentially embedded in paraffin wax blocks using rotary microtome. Tissue sections of 3–5 μm thick were cut and prepared on clean slides for Hematoxylin–Eosin (H–E) staining before mounting in neutral DPX medium. Prepared slides were examined at 400x magnification by trained pathologist.

### 2.7 Statistical analysis

All statistical analyses were conducted with Graphpad prism 5.0^®^ computer programs. One-way ANOVA was used to determine the differences (*p *< 0.05) among the various groups. Difference between each treatment group and the negative control was determined using Dunnett multiple post-hoc test (DMPT) procedure at *p* < 0.05.

## 3 Results

### 3.1 Heavy metal and physico-chemical characterization of the effluent

Heavy metals and physico-chemical parameters of the effluent are presented in Table 1. Fe, Mn and Cu concentrations in the sample were higher than national and international allowable limits. Cd, As, Ni and Cr were below detectable limits. The pH value is within normal range for effluents both in national and international allowable standards. COD, BOD, phosphate, alkalinity and total dissolved solids were above permissible limits while all other analyzed parameters were below permissible limits.

**Table 1:** Physico-chemical characteristics and heavy metals detected in the pharmaceutical effluent and national and international permissible standards.

Parameters	Effluent	NESREA^a^	USEPA^b^
pH	6.3	6.0–9.0	6.5–8.5
Nitrate	3.50	10	10
Ammonia	0.13	10	0.02
BOD^c^	4.60	50	–
COD^d^	93.0	90	–
Phosphate	2.25	2.0	–
Chloride	16.0	250	250
Sulphate	31.0	250	250
Hardness	56.0	–	0–75
Alkalinity	405	150	2.0
TDS^e^	107.0	–	–
Cu	0.99	0.5	1.3
Fe	0.60	–	0.3
Cd	BDL	9.02	0.05
Mn	0.30	0.02	0.05
As	BDL	–	0.01
Ni	BDL	0.05	–
Cr	BDL	0.05	0.1

*All values are in mg/L except pH. BDL = below detectable limit.

a National Environmental Standards and Regulations Enforcement Agency (2009) Nigeria) maximum permissible limits for wastewater discharge.

b United State Environmental Protection Agency (2006). www.epa.gov/safewater/mcl.html

c Biochemical Oxygen Demand.

d Chemical Oxygen Demand.

e Total Dissolved Solid.

### 3.2 Biochemical indicators of hepatic function, lipid peroxidation and oxidative stress

**Fig. 1:**
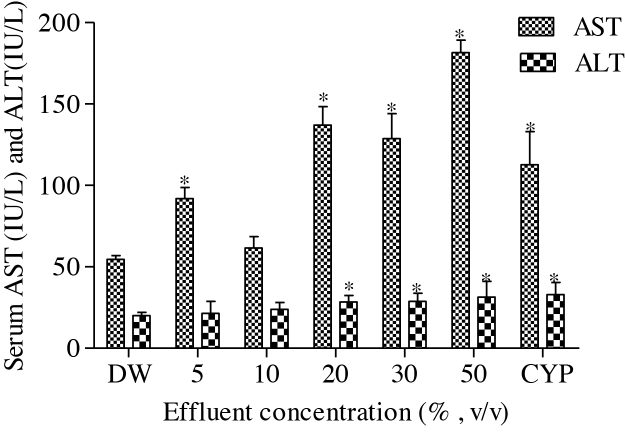
Effects of pharmaceutical effluent on serum ALT and AST activities. End point represents mean ± SD for 5 rats. Values are significantly different *p < 0.05 compared to the corresponding negative controls. DW – Distilled Water; CYP – Cyclophosphamide (40 mg/Kg bwt).

Results of the antioxidant enzyme activities and lipid peroxidation in the effluent treated rats; SOD and CAT activities, and MDA concentration significantly (*p* < 0.05) increase compared to the negative control. The fold increase in SOD (2.44, 2.34, 3.82, 6.03 and 5.25), CAT (1.71, 1.07, 1.07, 6.79 and 7.43) and MDA (1.97, 1.48, 1.26, 1.70 and 5.52) according to the effluent concentrations; 5, 10, 20, 30 and 50%, respectively, was not concentration dependent (Table 2). Serum hepatic function tests: ALT and AST activities, and direct and total bilirubin concentrations and serum renal function test: creatinine concentration significantly (*p* < 0.05) increase in the effluent treated rats compared to the negative control (Fig. 1 and Table 2). The fold increase in AST (1.69, 1.13, 2.51, 2.36 and 3.33), ALT (1.07, 1.20, 1.41, 1.44 and 1.57), DB (1.09, 1.27, 1.82, 1.18 and 1.18), TB (1.65, 1.82, 2.82, 1.41 and 1.24) and creatinine (1.12, 1.31, 1.80, 1.33 and 1.19) according to the effluent concentrations; 5, 10, 20, 30 and 50%, respectively, were not concentration dependent for all the tested parameters.

**Table 2:** Effects of pharmaceutical effluent on serum biochemical parameters in Wistar rats.

Conc (%)	SOD (U/mg)	CAT (μmol/mg)	MDA (nmol/ml)	TB (mg/dL)	DB (mg/dL)	Creatinine (mg/dL)
DW	5.52 ± 0.90	0.14 ± 0.02	5.57 ± 1.35	0.17 ± 0.04	0.11 ± 0.02	0.81 ± 0.17
5	13.48 ± 8.57	0.24 ± 0.33*	10.95 ± 1.96*	0.28 ± 0.04*	0.12 ± 0.02	0.91 ± 0.12
10	12.89 ± 3.98	0.15 ± 0.01	8.27 ± 0.69	0.31 ± 0.07*	0.14 ± 0.05	1.06 ± 0.20*
20	21.08 ± 1.15*	0.15 ± 0.01	7.03 ± 0.34	0.48 ± 0.21*	0.20 ± 0.07	1.46 ± 0.08*
30	33.31 ± 5.96*	0.95 ± 0.31*	9.47 ± 4.82*	0.24 ± 0.11	0.13 ± 0.05	1.08 ± 0.21*
50	28.97 ± 4.98*	1.04 ± 0.33*	30.77 ± 6.80*	0.21 ± 0.05	0.13 ± 0.04	0.96 ± 0.08
CYP	5.96 ± 1.45	0.21 ± 0.04*	10.98 ± 2.47*	0.38 ± 0.04*	0.17 ± 0.02	0.91 ± 0.06

End point represents mean ± SD for 5 rats. Values are significantly different; *p < 0.05; compared to negative control. SOD (superoxide dismutase), CAT (catalase), MDA (malondialdehyde), TB (total bilirubin), DB (direct bilirubin). DW (Distilled Water); CYP (Cyclophosphamide (40 mg/Kg bwt).

### 3.3 Hematological analysis

The results of the hematological indices showed that RBC, HCT, HGB, MCH, PLT and WBC were reduced compared to the negative control. While this reduction was insignificant (*p* > 0.05) for RBC, HCT, HGB and MCH, it was marginally significant (*p* < 0.05) for PLT and WBC. Only MCHC increased in the effluent treated rats but was insignificantly (*p* > 0.05) different from the negative control (Table 3).

**Table 3:** Hematological profile of rats treated with pharmaceutical effluent.

Conc (%)	RBC (x10^6^ μL)	HCT (%)	HGB (g/dL)	MCH (pg)	MCHC (g/dL)	PLT (x10^6^ μL)	WBC (x10^3^ μL)
DW	7.22 ± 0.59	44.74 ± 2.23	11.16 ± 0.80	15.78 ± 0.24	23.24 ± 3.45	807.6 ± 119.0	11.92 ± 2.13
5	6.90 ± 0.39	43.96 ± 1.94	10.90 ± 0.48	15.68 ± 0.27	24.80 ± 0.53	551.8 ± 143.2	11.12 ± 2.44
10	6.81 ± 1.02	42.20 ± 7.74	10.62 ± 0.64	15.50 ± 0.19	24.36±1.11	510.6 ± 199.3	8.94 ± 1.66*
20	6.80 ± 0.45	42.92 ± 2.22	10.42 ± 0.86	14.98 ± 0.71	24.92 ± 0.62	533.8 ± 228.0*	8.98 ± 1.44*
30	6.85 ± 0.46	40.96 ± 2.71	10.38 ± 0.77	14.82 ± 0.37	25.34 ± 0.52	486.8 ± 178.3*	7.84 ± 1.96*
50	6.63 ± 0.15	39.78 ± 1.02	9.82 ± 0.30	14.32 ± 1.84	24.70 ± 0.64	435.5 ± 119.3*	7.74 ± 2.03*
CYP	6.65 ± 0.53	41.54 ± 2.35	9.62 ± 0.78	15.16 ± 0.61	24.26 ± 1.26	435.2 ± 176.4*	5.00 ± 2.85*
	(*p *= 0.514)	(*p* = 0.3782)	(*p* = 0.0637)	(*p* = 0.7658)	(*p* = 0.4495)	(*p* = 0.0313)	(*p* = 0.0454)

End points represent mean ± SD for 5 rats. RBC (Red blood cell count); HGB (Hemoglobin concentration); HCT (Percentage Hematocrit); PLT (platelets); WBC (White blood cell count); MCH (mean corpuscular hemoglobin); MCHC (mean corpuscular hemoglobin concentration); DW (Distilled water); CYP (Cyclophosphamide 40 mg/kg). Superscripts differ significantly (*p < 0.05) from corresponding control using Dunnett's multiple post hoc test. DW – Distilled water.

### 3.4 Micronucleus analysis

**Fig. 2:**
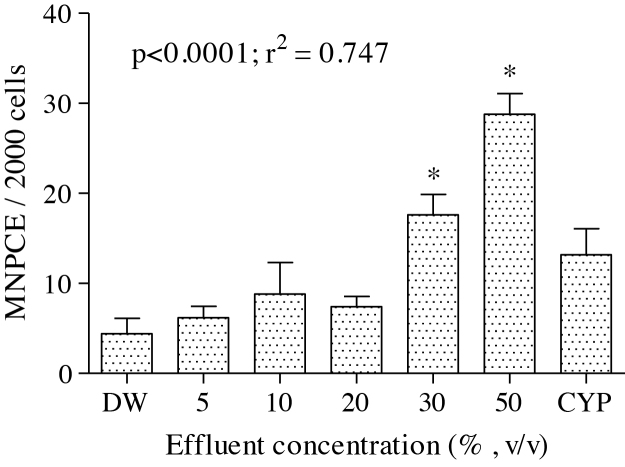
Effects of pharmaceutical effluent on micronuclei polychromatic erythrocyte formation (MNPCE). End point represents mean ± SD for 5 rats. Values are significantly different *p < 0.05 compared to the corresponding negative controls. DW – Distilled Water; CYP – Cyclophosphamide (40 mg/Kg bwt).

There was significant increase (*p* < 0.05) in the frequencies of micronucleated polychromatic erythrocyte formed in the bone marrow cells of the treated rats (Fig. 2). The effluent treatment concentrations; 5, 10, 20, 30 and 50 % induced 1.41, 2.0, 1.68, 4.0 and 6.55 folds increase respectively compared to the negative control.

### 3.5 Histopathological assessment of the liver and kidney

**Fig. 3:**
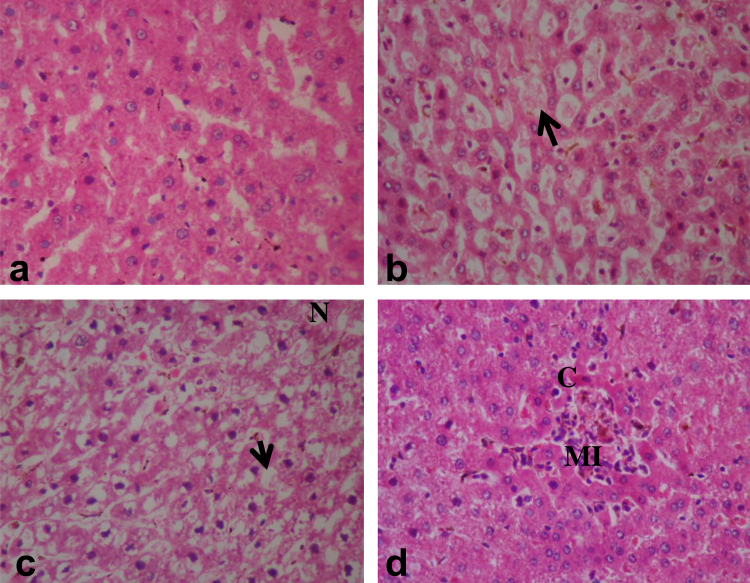
Sections of the liver from rat exposed to pharmaceutical effluent (H&E, ×400). (a) liver from rat in the negative control group showing apparently normal hepatocytes. (b) thinning of the hepatocytic cord with moderate kuffer cell hyperplasia (arrow). (c) Vacuolations of the hepatocytes (arrow) and necrosis (N). (d) Multifocal inflammatory cellular (MI) and vacuolar changes in the hepatoctes along with congestion of the central vein (C).

**Fig. 4:**
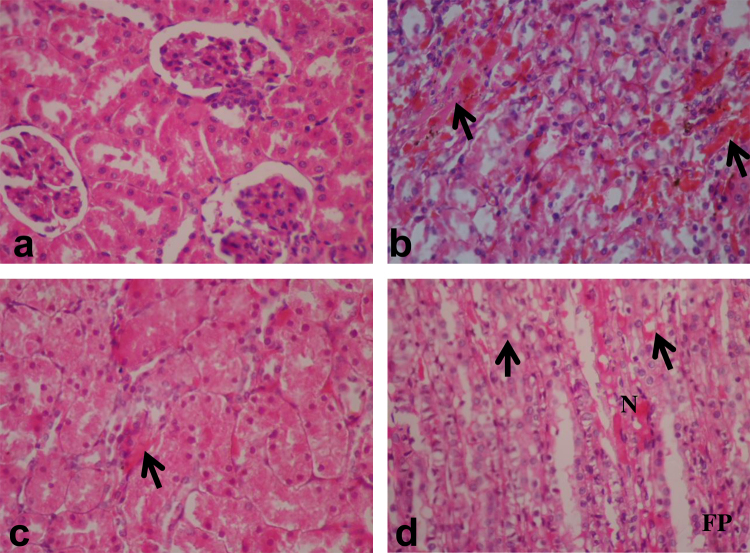
Sections of the kidney from rats exposed to pharmaceutical effluent (H&E, ×400). (a) kidney from a negative control rat showing apparently normal cortical and medullary section of the nephron. (b) Marked congestion of the renal blood vessels (arrow). (c) Swollen epithelium with congestions of the renal interstitium (arrow). (d) Vacuolar changes of the tubular epithelium cells (arrow) and fibroplastic proliferation (FP).

Fig. 3a–d presents the histopathological sections of the liver from the treated and control rats. Section of the liver from the negative control rats showed apparently normal pentagonal sections of the hepatocytes with a large spheroidal nucleus (Fig. 3a). Histopathology of the liver from effluent treated rats showed thinning of the hepatocytic cords (cord atrophy) with moderate kuffer cell hyperplasia (Fig. 3b). Also numerous and well spread vacuolations and necrosis of the hepatocytes were common among the 30 and 50% treated rats (Fig. 3c). Multifocal inflammatory cells and congestions of the central vein were also observed (Fig. 3d). Fig. 4(a–d) presents the histopathological sections of the kidney from effluent treated and control rats. Apparently normal histopathology of the cortex and medulla was observed in the kidney section of the negative control rat while some nephrotoxic lesions were observed in the treated rats. These lesions include marked congestions of the interstitial tissues (Fig. 4b), swollen epithelium along with congestions of the renal interstitium (Fig. 4c). Vacuolar and necrotic changes of the tubular epithelium cells and fibroplastic proliferation were also observed in the kidney of the effluent treated rats (Fig. 4d). The frequency of the severity of histopathological lesions in liver and kidney were increased according to the effluent concentrations in the exposed rats (Table 4).

**Table 4:** Severity of histological lesions in the liver and kidney of rats exposed to the pharmaceutical effluent.

Concentrations (%)	NC	5	10	20	30	50
Observed histological changes						
LIVER						
Hepatic vacuolation	−	+	+	++	+++	+++
Thinning of hepatocytic cords	−	–	–	−	+	++
Kuffer cell hyperplasia	−	–	+	−	+	++
Multifocal inflammation	−	+	−	−	+++	++
Congestion of hepatic blood vessels	−	−	−	++	++	+++
Necrosis	−	+	+	+	++	++

KIDNEY						
Congestion of renal blood vessels	−	+	+	+	++	+++
Swollen tubular epithelium	−	−	−	−	+	++
Necrotic changes	−	−	−	++	++	++
Renal vacuolar change	−	+	+	+	++	+++

NC – Negative control (distilled water).

Severity of the liver and kidney histological changes were assessed using the following scale:

−(change that was either absent or sporadic in all rats of a group).

+(a change that was found in a few animals of a group).

++(a change that was relatively common in all animals of a group).

+++(a change that was very often found in all animals of a group).

## 4 Discussion

The analyzed heavy metals in the effluent, except Zn, Mn, Fe and Cu, were lower than acceptable limits by national and international regulatory authorities (Table 1), was not in agreement with previous studies in which the concentrations of toxic metals; Cd, Cr, Pb, Ni, Zn and Cu, in nine pharmaceutical effluents from Lagos, Nigeria [1] and a pharmaceutical effluent from Lote-Pershuram industrial area, India [32], were above WHO recommended maximum concentration level. It is noteworthy that studies have reported high concentrations of toxic metals and organic compounds at varying concentrations above standard permissible limits in pharmaceutical effluents [1], [3], [4], [5], [32].

Liver and kidney in mammalian system are the most sensitive predictor of chemical toxicity that correlates well with histopathology and serum biochemistry with little inter-animal variations [33]. As the major site of chemical transformation, these organs are prone to the toxic effects of chemicals. When cell membranes of the hepatocytes are damaged, a number of cytoplasmic enzymes are released into the circulatory system providing basis for clinical diagnosis. Serum AST and ALT are the most used biochemical markers of hepatocellular necrosis and are considered sensitive indicators of hepatic chemical induced injuries [34]. Significant increase in serum ALT and AST activities and concomitant increase in total and direct bilirubin concentrations in the treated rats suggest acute hepatocellular damage due to the induction of necrosis by the xenobiotics in the effluent. Increase in lipid peroxidative damage induced by the toxic metals and possibly unanalyzed organic constituents of the effluent on the hepatocytes (necrosis), increased cell membrane permeability to ALT and AST from the cytoplasm into the blood circulation. Hepatic necrosis of the liver cells and significant increase in serum MDA in the treated rats are in support of this assertion. Serum creatinine concentration is a biomarker of renal injury and the elevation of this biomarker is usually associated with impairment of renal function [35]. Increase in serum creatinine in the treated rats compared to the negative control suggests kidney damage possibly due to the depression of glomerular filtration rate and renal tubular cell injury by the xenobiotics present in the effluent. The reports that e-waste leachate treated mice [16] and landfill leachates treated rats [15] elicited significantly high concentrations of serum transaminases and creatinine along with the induction of histopathological lesions are in concert with the observations herein.

Histological study provides the most reliable information on the type of lesions induced by chemicals in tissues. It is also useful in providing information on the toxic effects of xenobiotics that may not lead to alterations in biochemical parameters [36]. The histopathological lesions observations in the liver (Fig. 3) and kidney (Fig. 4) of rats exposed to sub-lethal concentrations of the pharmaceutical effluent, corroborates the biochemical findings suggesting liver and kidney dysfunctions induced by xenobiotics in the effluent [15]. The physico-chemical parameters analyzed in the effluent are known to modify metal toxicity and bioavailability [50]. It is possible that the high COD, TDS and more importantly alkalinity of the effluent modified toxicity of the metals (even at their low concentrations) leading to the observed genotoxicity and systemic toxicity [51]. It is also known that the metals and possibly organic compounds (though not analyzed) in the effluent acted in synergy and or antagonism to induce alterations in enzyme biochemistry, biological molecules and cell membrane [16], [37]. These alterations may be attributed to increased cellular formation of oxidative stress via creation of imbalance between reactive oxygen species (ROS) and the antioxidant systems [38]. SOD activity is important in preventing oxidative damage by scavenging and converting superoxide anions to hydrogen peroxides, while catalase causes the decomposition of the hydrogen peroxide to protect tissues from the actions of the highly reactive hydroxyl radicals [34]. During ROS production, the activities of these enzymes may be altered leading to the induction of pathological disorders and DNA damage [39]. The significant increase in the activities of SOD and CAT in the treated rats suggests harmful effects of the constituents, mostly the toxic metals, via excess undetoxified free radical formation. These unscavenged free radicals similarly induced lipoperoxide formation which caused lipid peroxidation in the cell membrane (increase MDA concentration) of the treated rats compared to the negative control. Li et al. [40] similarly reported alterations in SOD and CAT activities with concomitant change in MDA concentrations in landfill leachate treated mice due to the toxic metals of the leachate generated free radicals by autoxidation.

Hematological testing in rodents during toxicity and safety evaluations is generally acknowledged as integral part of safety assessment [30]. The tested effluent induced reduction in RBC, HCT, HGB, MCV, MCH, PLT and WBC and increased MCHC concentrations in the blood of the treated rats compared to the negative control, suggests the hematotoxic effects of the constituents of the effluent. It is suggested that hematological changes are associated with hematopathology and data from *in vivo* toxicological studies in mammals is one of the most predictive measures for human risk assessment [41]. The observed hematological alterations may indicate the effects of individual or interactive actions of the xenobiotics in the effluent on the hematopoietic tissues in the bone marrow of the treated rats. This assertion is supported by the reports that xenobiotics in landfill leachates [14], distillery soil leachate [42] and textile dye effluent [43] induced significant alterations in hematological parameters of rats and mice, was due to the disturbance in the hematopoiesis of the bone marrow system.

MN test is the most widely utilized test for the genotoxic and mutagenic assessment of xenobiotics due to its technical simplicity, less time consumption and ability to detect both clastogens and aneugens [44]. Micronuclei (MN) are formed in addition to the main nucleus in cells as a result of acentric fragments or lagging chromosomes that failed to incorporate into either of the daughter nuclei during cell division [44]. The significant increase in MNPCE observed in the bone marrow cells of the effluent exposed rats showed that the components of the effluent are genotoxic, leading to DNA damage. Xenobiotics in pharmaceutical effluents are known mutagens and carcinogens that are capable of inducing chromosomal damage may increase genomic instability. Mn and Fe, observed in the effluent, though are considered essential elements that play roles in normal enzyme activities that enhance cell growth, but when in high concentrations due to chronic low dose exposure, may provoke DNA damage by binding to biologically sensitive molecules; DNA or forming dangerous free radicals [45]. The reports that pharmaceutical effluent induced MNPCE and abnormal sperm morphology in mice [8], (2009), and chromosome aberrations in *Allium cepa* and mice [8], [22] are in agreement with the findings herein and further supports that xenobiotics in the effluents are clastogens. The report of Alexandrov et al. [46] that Al and Fe interacted synergistically to induce DNA damage in human neural (HN) cells via oxidative stress generation, further supports the findings herein that the components of the effluent probably acted in synergy to elicit free radical formation which induced the observed MNPCE formation in the treated rats.

There are increasing reports on the role of oxidative stress elicited by mixture of xenobiotics present in leachates and effluents on the induction of abnormal cellular functions and pathological disorders [16], [47]. Oxidative stress induced cell damage may occur via a number of mechanisms [48] and the damaged cells may be eliminated either by programmed cell death (apoptosis) or accidental cell death (necrosis). Effluent induced cellular necrosis and inflammation in the liver and kidney cells of the treated rats may be associated with the destruction of certain signal pathways in the cells and the disruption of mitochondrial functions via inflammatory process [49]. This is the first reports that showed that pharmaceutical effluent altered SOD and CAT activities, and MDA concentration in rats. The induced alterations correlated with cellular pathological lesions in the hepatocytes and kidney tubules, altered hematological indices, and increased MNPCE formation in the bone marrow cells of treated rats. In conclusion, pharmaceutical effluent induced somatic mutation and systemic toxicity in rats is associated with oxidative stress induction.

## Conflict of interests

The authors declared that there is no conflict of interest.
